# Hybrid peptide NTP-217 triggers ROS-mediated rapid necrosis in liver cancer cells by induction of mitochondrial leakage

**DOI:** 10.3389/fonc.2022.1028600

**Published:** 2023-01-12

**Authors:** Hao Yin, Xingyan Fu, Hanyu Gao, Han Gao, Yannan Ma, Xitong Chen, Xueqi Zhang, Shan-Shan Du, Yun-Kun Qi

**Affiliations:** ^1^ School of Pharmacy, Qingdao University Medical College, Qingdao University, Qingdao, China; ^2^ School of Stomatology, Jining Medical University, Jining, China; ^3^ Department of Gynecology, Qilu Hospital, Shandong University, Jinan, China; ^4^ College of Chemical Engineering, Qingdao University of Science and Technology, Qingdao, China

**Keywords:** hybrid peptides, liver cancer, mitochondria, ROS, necrosis, apoptosis

## Abstract

Liver cancer is the third leading cause of cancer-associated mortality globally, and >830,000 patients with liver cancer undergoing treatment succumbed to the disease in 2020, which indicates the urgent need to develop a more effective anti-liver cancer drug. In our previous study, nucleus-targeting hybrid peptides obtained from the fusion of LTX-315 and the rhodamine B group possessed potent anti-adherent cancer cell activity. Hybrid peptides accumulated in the cell nucleus and damaged the nuclear membrane, resulting in the transfer of reactive oxygen species (ROS) from the cytoplasm to the nucleus and the induction of apoptosis. However, the source of the high concentration of ROS within the cytoplasm is unclear. Moreover, although our previous study demonstrated that hybrid peptides possessed potent anticancer activity against adherent cancer cells, their efficacy on liver cancer remained unexplored. The current study found that the hybrid peptide NTP-217 killed liver cancer cells after 4-h treatment with a half-maximal inhibitory concentration of 14.6-45.7 μM. NTP-217 could stably accumulate in the liver tumor tissue and markedly inhibited liver tumor growth in mice. Furthermore, NTP-217 destroyed mitochondria and induced the leakage of mitochondrial contents, resulting in the generation of a substantial quantity of ROS. Unlike the apoptosis induced by 24 h of treatment by NTP-217, 4 h of treatment caused ROS-mediated necrotic cell death. These findings suggested that short-time treatment with hybrid peptides could trigger ROS-mediated rapid necrosis in liver cancer cells, and provided a basis for the future development of hybrid peptides as anti-liver cancer agents.

## Introduction

Liver cancer remains a leading public health problem worldwide, and the data from the International Agency for Research on Cancer indicated that >905,000 individuals were diagnosed with liver cancer globally, and it has been rated as the third leading cause of death associated with cancer in 2020 (1). Since in the early stage of liver cancer patients are generally asymptomatic, they are usually diagnosed at an advanced stage (2), when the tumor is not amenable to surgical resection (3); thus, pharmacotherapy is an important alternative for the treatment of advanced liver cancer. Sorafenib and lenvatinib, the only two drugs for advanced liver cancer approved by the Food and Drug Administration, showed promising therapeutic activity and few toxic side effects (4). Remission is difficult to sustain, and patients are unlikely to have lasting benefits from target drugs due to acquired drug resistance (5). Therefore, there is an urgent need to develop more effective anti-liver cancer drugs.

Peptide drugs including peptide-drug conjugates play important roles in cancer treatment since they possess various biological activities, including membranolysis, inhibition of angiogenesis, inhibition of programmed cell death (PD)-1/PD-ligand 1 interaction, and mitochondrial damage (6–11). The oncolytic peptide LTX-315, derived from bovine lactoferricin, exerts anticancer effects *via* membranolysis and induced immune response (6). To increase its anticancer activity, hybrid peptides from the fusion of LTX-315 and the rhodamine B group were designed and synthesized (12). Compared with that of LTX-315, hybrid peptides had an increased anticancer activity by 2.4-37.5-fold in different adherent cancer cell lines. Unlike LTX-315, which targets mitochondria, hybrid peptides accumulate in the cell nucleus and damage the integrity of the nuclear membrane, leading to the accumulation of reactive oxygen species (ROS) from the cytoplasm into the nucleus, thus resulting in the induction of DNA double strand break (DSB)-associated apoptosis (12). However, upon treatment with hybrid peptides, the source of ROS is elusive, which needs to be investigated.

Increasing ROS levels is an effective method to kill cancer cells (13). Numerous studies have documented that certain anticancer agents are able to evoke the autophagic cell death of hepatocellular carcinoma cells by enhancing the level of ROS (14–16). For example, Li et al. (17) reported that tetrandrine triggered mitochondrial dysfunction and ROS accumulation in hepatoma cells, leading to autophagic cell death. Since hybrid peptides also increase the production of ROS, these peptides may possess potential anti-liver cancer activity. In addition, LTX-315 has been shown to induce the death of lymphoma cells within 4 h (18). Whether hybrid peptides can induce rapid cell death (4 h) remains unknown. Hence, the short-time anticancer effects of hybrid peptides against hepatoma are worth investigating.

In the present study, the representative hybrid peptide NTP-217 was used to investigate its potential rapid anti-liver cancer activity both *in vitro* and *in vivo*. Since mitochondria are the main source of intracellular ROS (19), the effects of NTP-217 on mitochondria were examined. As 4-h treatment may not be sufficient to induce apoptosis, the associations between NTP-217 and necrosis or apoptosis were further investigated.

## Materials and methods

### Chemical synthesis of NTP-217

The hybrid peptide NTP-217 was synthesized by the DIC/Oxyma-based accelerated solid-phase peptide synthesis strategy (20–22). To be specific, 160 mg (0.05 mmol, 1 eq) of Rink Amide AM resin (Tianjin Nankai HECHENG S&T, loading: 0.32 mmol g^−1^) was placed in the peptide synthesis vessel and swelled in 8 ml of the DMF/DCM (1:1, v/v) solution for 3 h in a constant temperature shaker at 28°C. The first Fmoc protecting group was removed by the 20% (v:v = 1:4) piperidine/DMF solution (twice: first 5 min and then 10 min) at 28°C. Then, the resin was thoroughly washed by DMF and DCM. The C-terminal amino acid residue was coupled twice (15 min and then 25 min), utilizing the DMF solution of the Fmoc-amino acid (3 eq), DIC (6 eq), and Oxyma (3 eq) at 50°C. It should be pointed out that the Fmoc-amino acid was activated for about 5 min at room temperature before adding to the resin. Generally, double coupling (first 10-15 min and then 25-35 min) is enough for the following amino acids. The Kaiser test was used to detect the completion of the coupling step. 20% piperidine/DMF solution was used to remove the following Fmoc groups at 50˚C (twice: first 3 min and then 6 min).

After the coupling of the last amino acid residue and the 4-aminobutanoic acid group, the double coupling (40 min and then 60 min) at 28°C was conducted for the ligation of the N-terminal rhodamine B, utilizing the DMF solution of rhodamine B (3 eq), HATU (2.7 eq), HOAT (3 eq), DIPEA (6 eq). The rhodamine B should be activated for about 60 sec at room temperature before adding to the resin. After the coupling of the N-terminal rhodamine B, the resin was dried and then treated with the peptide cleavage cocktail (TFA/phenol/water/TIPS, 88:5:5:2, v/v/v/v) for 2.5 h at 28°C. Then, the combined filtrate was concentrated by blowing with pure nitrogen. Subsequently, the crude peptide was dissolved in H_2_O/CH_3_CN and purified by the semi-preparative RP-HPLC. Finally, the solid target peptide (NTP-217) with a purity above 96% was obtained by freeze-drying.

### Cell culture

The human liver cancer cell lines HepG2, Hep3B, Hep3B-Luc2-tdT, and PLC/PRF/5 were purchased from the National Infrastructure of Cell Line Resource (Beijing, China) and maintained in MEM (Gibco) supplemented with 10% fetal bovine serum (FBS). HuH7 and HCCLM3 obtained from the National Infrastructure of Cell Line Resource as well as Hepa 1-6 (Beyotime Biotechnology) were cultured in DMEM (Gibco) with 10% FBS. All cells were cultured in a humidified atmosphere of 5% CO_2_ at 37°C.

### Cell proliferation assay

The peptide LTX-315 was synthesized and provided by KS-V Peptide Biological Technology Co., Ltd. (Hefei, China), while sorafenib was obtained from Shanghai Aladdin Biochemical Technology Co., Ltd. The effect of NTP-217 on the proliferation of hepatoma cells was assessed by MTT assay, as previously described (3). Briefly, liver cancer cells were seeded at a density of 10,000 cells/well in a 96-well plate and adhered overnight. Stock solutions (30 mM) dissolved in DMSO were diluted to various concentrations (1.5625, 3.125, 6.25, 12.5, 25 and 50 μM for NTP-217, or 1, 3, 10, 30, 100 and 300 μM for LTX-315 and sorafenib). After exposure to the indicated drugs for 4 h at 37˚C, 15 μl MTT solution (5 mg/ml; Shanghai Aladdin Biochemical Technology Co., Ltd.) was added for 4 h, and the produced formazan was then dissolved with 150 μl DMSO. Optical density (OD) was measured with a microplate reader (Tecan Group, Ltd.) at 490 nm. The inhibition rate was calculated with the following equation: Inhibition rate = (OD_control_ - OD _experimental group_)**/**(OD _control_- OD _blank_) × 100%. The half-maximal inhibitory concentration (IC_50_) was calculated with SPSS 22 software (IBM Corp).

### Cell migration assay

Hepa 1-6 cells in the logarithmic growth phase were digested with trypsin and inoculated into a 6-well plate at a cell density of 4x10^5^ cells/well. When the fusion rate was close to 90%, cells were scratched with the tip of a sterile pipette. The scratched cells were washed twice with PBS and then treated with a low-serum (1%) medium containing different concentrations of drugs for 12 or 24 h. Images were viewed by microscopy (Ti-S, Nikon Corporation) and quantified with ImageJ software version 1.53c (National Institutes of Health).

### *In vivo* imaging analysis

C57 mice with Hepa 1-6 hepatoma at the right flank were anesthetized with chloral hydrate (0.35 ml/100 g, 10%, intraperitoneal injection). Subsequently, 50 μl NTP-217 (10 mg/ml) or 50 μl LTX-315 (10 mg/ml) were injected into tumors, and imaging was performed as indicated times. All fluorescent images were carried out *via* an IVIS^®^ Spectrum small animal imaging system (PerkinElmer), and excitation wavelength was 570 nm and emission wavelength was 620 nm.

### Animal studies

The experimental protocol was approved by the Medical Ethics Committee of the Affiliated Hospital of Qingdao University, and all the applicable institutional and governmental regulations concerning the ethical use of animals were followed. A total of 18 male C57 mice (18-20 g) of 4-6 weeks of age, were purchased from Beijing Vital River Laboratory Animal Technology Co., Ltd. Hepa 1-6 cells (5x10^7^ cells/ml) suspended in DMEM (Beijing Solarbio Science & Technology Co., Ltd) without phenol red were injected subcutaneously (200 μl) into the flank of mice (23). Tumor size was measured with a caliper every other day, and the volume was calculated with the following formula: Volume = 0.5 x (length) x (width)^2^. When the tumor volume reached ~100 mm^3^, the mice were randomly divided into 3 groups (n=6/group), as follows: i) Control group (normal saline, intralesional injection); ii) NTP-217 group (50 μl at 10 mg/ml, intralesional injection); and iii) LTX-315 group (50 μl at 10 mg/ml, intralesional injection). All peptide solutions were prepared by dissolving the peptides in normal saline prior to the experiments. When the volume of the tumor exceeded 2000 mm^3^ or presented ulcerations, mice were euthanatized by cervical dislocation. Upon observing cardiac arrest, the tumor tissue was resected and weighed.

### Histology

Mice with Hepa 1-6 tumors were administrated intralesionally normal saline, 0.5 mg LTX-315 (50 μl* 10 mg/ml) or 0.5 mg NTP-217 (50 μl* 10 mg/ml) for continuous 3 days, respectively. 1 day later they were sacrificed and tumors were harvested carefully and fixed in 4% PFA. Samples were embedded in paraffin and cut into 6-μm thick slices. Slices were dewaxed and stained by hematoxylin/eosin dye (Solarbio). Images were acquired using a microscope (Nikon).

### Western blotting

Hepa 1-6 cells reached confluence in 6-well plates and were then incubated with different concentrations of NTP-217 for 4 h. Next, the cells were washed twice with cold PBS and then lysed using RIPA lysis buffer (Thermo Fisher Scientific, Inc.) with phosphatase inhibitor cocktail I (cat. no. C0002; TargetMol) and Protease Inhibitor Cocktail (cat. no. C0001; TargetMol). Protein concentration was measured with BCA Protein Assay Reagent Kit (Thermo Fisher Scientific, Inc.). Samples (30 μg/lane) were subjected to 12% SDS-PAGE and then transferred to PVDF membranes (Millipore Sigma). The membranes were blocked with QuickBlock™ Blocking Buffer for Western Blot (Beyotime Institute of Biotechnology) and then incubated overnight at 4˚C with primary antibodies against Poly [ADP-ribose] polymerase 1 (1:5,000; cat. no. 66520-1; ProteinTech Group, Inc.), caspase-3 (1:10,000; cat. no. ab32499; Abcam), cleaved caspase-3 (1:1,000, cat. no. 9661, Cell Signaling Technology, Inc.) and GAPDH (1:10,000; cat. no. abs132004; Absin). The membranes were washed with TBS with 0.05% Tween-20 (TBST) buffer thrice for 5 min each and incubated with a secondary antibody (1:10,000; cat. no. abs20002; Absin) for 1 h at room temperature. Protein bands were visualized by using an ECL reagent (Thermo Fisher Scientific, Inc.) and detected with ECL Western Blotting Detection System (Bio-Rad Laboratories, Inc.).

### Measurement of intracellular ROS levels

Intracellular ROS levels were measured using the fluorescent probe dichloro-dihydro-fluorescein diacetate (DCFH-DA; Beyotime Institute of Biotechnology). DCFH-DA is easily oxidized to fluorescent DCF by intracellular ROS, and therefore, the fluorescent intensity of DCF reflects the ROS level. Briefly, Hepa 1-6 cells cultured in 6-well plates were harvested using trypsin-EDTA (Thermo Fisher Scientific, Inc.) and exposed to various concentrations of NTP-217 for 1 h. Subsequently, the cells were incubated with 10 μM DCFH-DA for 20 min at 37˚C and then washed 3 times with DMEM without phenol red. Next, the cells were transferred to 96-well black microplates, and the fluorescence intensity was measured at 488-nm excitation and 525-nm emission with FlexStation 3 Multi-Mode Microplate Reader (Molecular Devices, LLC).

### Cell apoptosis assay

The apoptotic cell was detected by Hoechst 33258 and Greennuc™ caspase-3 assay kit (Beyotime). GreenNuc™ Caspase-3 Substrate would be a green fluorescent molecule after it is activated by caspase-3. Briefly, Hepa 1-6 cells seeded in 24-well plates were incubated with the different concentrations of NTP-217 for 4 h and stained with 5μM GreenNuc™ Caspase-3 Substrate and Hoechst 33258 (Invitrogen, 2 μg/ml) for 30 min. After washing twice with PBS, images were immediately viewed by fluorescence microscopy (Nikon)

### Determination of mitochondrial membrane potential (MMP)

The fluorescent dye JC-10 (Beijing Solarbio Science & Technology Co., Ltd.) was used to investigate if NTP-217 was able to change the MMP. The green, fluorescent JC-10 probe exists as a monomer at low membrane potentials, whereas at higher potentials, JC-10 forms red-fluorescent aggregates. Briefly, Hepa 1-6 cells were seeded in a 6-well plate, and after 12 h, they were incubated with 25 μM oncolytic peptides for different lengths of time. After treatment, the cells were incubated at 37˚C for 30 min with 200 nM JC-10, washed twice with PBS and placed in DMEM without phenol red. Lastly, images were visualized by fluorescence microscopy (Nikon Corporation), and the ratios of red/green fluorescent densities were calculated by ImageJ software version 1.53c (National Institutes of Health).

### Fluorescence leakage assay

Hep3B-Luc2-tdT cells expressing tdTomato red fluorescent protein were seeded in a 96-well plate at a density of 1x10^4^ cells per well and cultured overnight. Subsequently, the complete medium was replaced with PBS containing different concentrations of LTX-315 or NTP-217. Three replicate wells were assayed for each concentration. After treatment for 0.5 h, the PBS in the wells was collected and centrifuged at 1,000 x g for 5 min at room temperature (~25˚C). The supernatant was then transferred to a black 96-well plate (Thermo Fisher Scientific, Inc.) and measured with FlexStation 3 Multi-Mode Microplate Reader (Molecular Devices, LLC) at 554/581 nm (excitation and emission wavelengths). The corresponding concentrations of NTP-217 were used as a background correction to remove the interference from NTP-217 fluorescence, while 1% Triton-X-100 treatment was used as a positive control for 100% leakage.

### ATP level assay

Hepa 1-6 cells were seeded in a 96-well plate at a density of 1x10^4^ cells per well and cultured overnight. Subsequently, the complete medium was substituted by DMEM without phenol red containing different concentrations (0, 3.125, 6.25, 12.5, 25 and 50 μM) of NTP-217. Three replicate wells were assayed for each concentration. After treatment for 4 h, the solution in the wells was collected and centrifuged at 1,000 x g for 5 min at room temperature. The supernatant was then transferred to a black 96-well plate (Thermo Fisher Scientific, Inc.) and 100 μl Celltiter-LUMI™ luminescence detection reagent (Beyotime Institute of Biotechnology) was added to each well. Subsequently, the plate was incubated at room temperature for 10 min to stabilize the luminescence signal. Chemiluminescence detection was then performed using FlexStation 3 Multi-Mode Microplate Reader (Molecular Devices, LLC) with 1 sec of detection time.

### Propidium iodide staining

PI was utilized to distinguish cells without intact cell membranes. Hepa 1-6 cells were seeded in 12-well plates and cultured for 12 h. Next, the cells were incubated with 2 μg/ml Hoechst 33258 (Invitrogen; Thermo Fisher Scientific, Inc.) for 30 min, and then the medium was substituted by 500 μl MEM containing 2 μg/ml PI (Shanghai Aladdin Biochemical Technology Co., Ltd.) plus 25 μM NTP-217, and incubated for different times. Upon washing the cells 3 times, observation by fluorescence microscopy was performed (Nikon Corporation).

### Cell fluorescence imaging

Hepa 1-6 cells were seeded in 12-well plates and adhered overnight. The complete growth medium was then substituted by 1 ml phenol red-free MEM containing 2 μg/ml Hoechst 33258 (Invitrogen; Thermo Fisher Scientific, Inc.) and 200 nM Mito-Tracker Green (Beyotime institute of Biotechnology). After 0.5-h treatment, the supernatant was discarded, and the cells were treated with 50 μM NTP-217 or 50 μM LTX-315 plus 50 μM rhodamine B for different lengths of time. Upon washing 3 times, images were obtained by fluorescence microscopy (Nikon Corporation).

### Statistical analysis

Unless otherwise indicated, data are described as the mean ± SD. Unless otherwise indicated, data were analyzed by one-way analysis of variance (ANOVA) followed by the Tukey test. The limit of statistical significance was P < 0.05. Statistical analysis was performed with Prism 7 for windows software (GraphPad Software, Inc).

## Results

### Proliferation and migration inhibition of liver cancer cells by NTP-217

**Figure 1:**
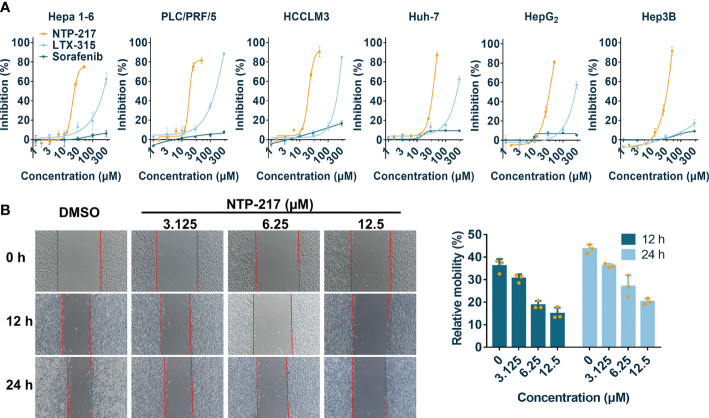
Cytotoxicity and anti-migration ability of NTP-217 in hepatoma cells. **(A)** Inhibition rates of hepatoma cells after treatment with NTP-217, LTX-315 or sorafenib at various concentrations (1.5625, 3.125, 6.25, 12.5, 25 and 50 μM for NTP-217, or 1, 3, 10, 30, 100 and 300 μM for LTX-315 and sorafenib) for 4 h (n=3). **(B)** Anti-migration effect of different concentrations of NTP-217 against Hepa 1-6 cells (left panel; objective magnification, x4) and quantification of the anti-migration ability (right panel). Data are expressed as the mean ± SD (n=3).

NTP-217 was prepared using solid-phase peptide synthesis and obtained with >96% purity (**Figures S1**, **S2**). MTT assay was conducted to analyze the effects of NTP-217, LTX-315 and sorafenib on the proliferation of various liver cancer cell lines. Although peptides can bind to and be neutralized by serum proteins, as drug treatment time is very short and only four hours, this assay was not performed in a serum-free medium. The commonly used molecular targeted drug sorafenib was utilized as a small molecule control. After 4 h of treatment, all drugs, with the exception of sorafenib, inhibited the proliferation of liver cancer cells in a dose-dependent manner (**Figure 1A**). Although LTX-315 induced the suppression of cell proliferation on hepatoma cells, this proliferation suppression was relatively weak (IC_50_>120 μM for 4 h). By contrast, the hybrid peptide NTP-217 appeared to have the most potent anti-liver cancer activity with IC_50_ = 14.6-45.7 μM, which increased 4.8-8.7-fold relative to LTX-315 (**Table 1**). Because Hepa 1-6 cells possess tumorigenicity in C57 mice, we also tested *in vivo* anticancer efficacy of NTP-217 in the Hepa 1-6 tumor model. To correspond to animal experiments, the subsequent research would be performed on Hepa 1-6 cells.

**Table 1:** Comparison of the half-maximal inhibitory concentration of NTP-217, LTX-315 and sorafenib on liver cancer cells for 4 h.

Agents	Sequences	Hepa 1-6	PLC/PRF/5	HCCLM3	Huh-7	HepG_2_	Hep3B
NTP-217	rhodamine B-GABA -KKWWKKW(Dip)K-NH_2_	21.6 ± 1.0	14.6 ± 1.1	22.4 ± 5.1	40.1 ± 1.3	48.8 ± 0.7	45.7 ± 1.7
LTX-315	H-KKWWKKW(Dip)K-NH_2_	147.2 ± 36.1	127.7 ± 20.0	121.0 ± 4.3	201.1 ± 10.8	237.0 ± 29.7	>300
Sorafenib		>300	>300	>300	>300	>300	>300

Data are presented as the mean ± standard deviation of 3 independent experiments.

To evaluate the anti-cell-migration activity of different concentrations of NTP-217, a wound scratch assay was performed against Hepa 1-6 cells (**Figure 1B**). It was found that the wound-healing speed was suppressed by all concentrations of NTP-217 in comparison with that of the DMSO-treated group. Quantification revealed that the cell mobility rate of the group treated with 12.5 μM NTP-217 was only 14.9% for 12 h, which was lower than that of other groups, demonstrating the efficacious anti-migratory activity of NTP-217.

### Continuous accumulation of NTP-217 in tumor tissue

**Figure 2:**
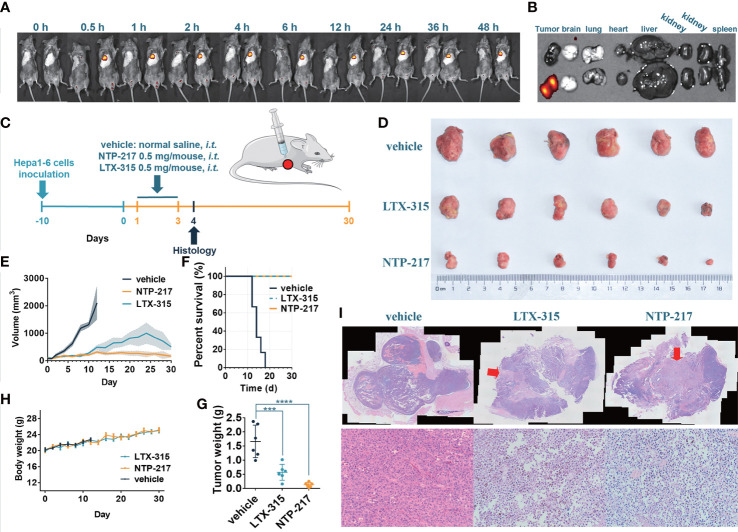
NTP-217 stably accumulates in tumor tissue and inhibits tumor growth. **(A)** *In vivo* fluorescence images of mice with Hepa 1-6 tumors were obtained at different times after intralesional injection (50 μl of 10 mg/ml) of (left) LTX-315 or (right) NTP-217. **(B)** Fluorescence images of the tumor and main organs of Hepa 1-6 tumor-bearing mice at 48 h post-injection of (top) LTX-315 or (bottom) NTP-217. **(C)** Schematic illustration of the timeline required for the therapeutic experiments. **(D)** Images of excised Hepa 1-6 tumors. **(E)** Tumor growth kinetics of different groups. mean ± SEM. **(F)** Survival analysis of Hepa 1-6 tumor-bearing mice. **(G)** Weight of Hepa 1-6 tumors. mean ± SD. ^***^P<0.001, ^****^P<0.0001. **(H)** Body weight of different groups. mean ± SD. **(I)** Representative (top) hematoxylin and eosin staining (objective magnification, x4) and (bottom) enlargement of the injection area (objective magnification, x10). The arrow indicates the injection area of oncolytic peptides. *i.t.*: intralesional injection.

To clarify whether NTP-217 would be suitable for liver cancer treatment *in vivo*, the distribution and stability of NTP-217 in the tumor within living mice were investigated. Imaging experiments were performed after intratumoral injection of 50 μl NTP-217 or LTX-315. Upon injection of LTX-315, no fluorescence was observed, whereas intratumoral injection of NTP-217 resulted in an intense fluorescence signal in tumor tissue (**Figure 2A**). Time-dependent imaging *in vivo* also indicated a stable level in the fluorescence responses in the tumor until 48 h post-injection, and there was no signal in other organs (**Figure 2B**). Living imaging in mice indicated that NTP-217 was capable of accumulation in tumor tissue with high selectivity, which provides a solid foundation for further tumor treatment *in vivo*.

### Growth inhibition of tumor by NTP-217

To examine the *in vivo* efficacy of NTP-217, mice bearing Hepa 1-6 tumor tumors were treated with NTP-217, LTX-315 or normal saline for 30 days (**Figure 2C**). As shown in **Figures 2D–F**, NTP-217 induced a significant inhibition of hepatoma growth. Intralesional administration of 0.5 mg NTP-217 or LTX-315 in the established tumors suppressed tumor growth by 92.0% or 65.6%, respectively (tumor weight; **Figure 2G**). By contrast, tumor growth in the normal saline-treated mice was not inhibited, and all animals were euthanized within 18 days (median survival time, 14 days; **Figure 2F**). NTP-217-treated, LTX-315-treated and control mice maintained a comparable body weight during drug treatment (**Figure 2H**). NTP-217 at the dose tested was well tolerated and did not cause any observable toxicity. When 1 mg LTX-315 was injected into tumors, it caused a focal necrotic skin injury, but 0.5 mg LTX-315 or NTP-217 did not cause such necrotic injury (data not shown). In addition, hematoxylin and eosin staining of the excised tumor tissue showed notable levels of necrosis in the NTP-217 and LTX-315 groups (**Figure 2I**).

### Non-apoptotic cell death induced by NTP-217

**Figure 3:**
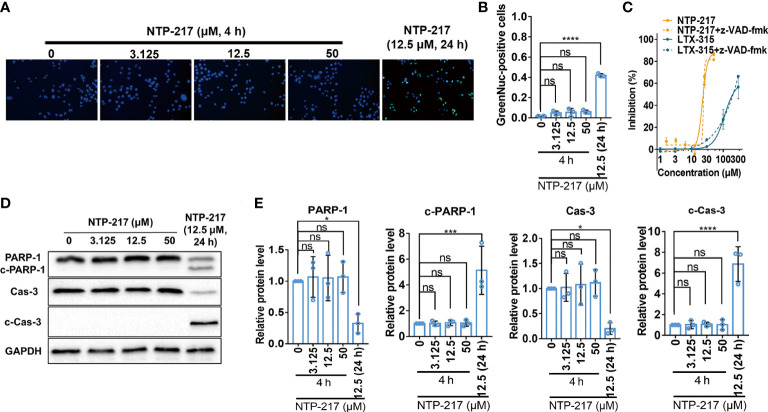
Failure of NTP-217 to induce caspase-3 activation and apoptosis. **(A)** Hepa 1-6 cells were treated with different concentrations of NTP-217 for 4 h or with 12.5 μM NTP-217 for 24 h. Cells were stained with a caspase-3/7 assay kit and Hoechst 33258 (objective magnification, x4). **(B)** Quantification of apoptotic cells by counting the ratio of GreenNuc-positive and Hoechst 33258-positive cells. **(C)** Hepa 1-6 cells were incubated with NTP-217 alone or in the presence of 20 μM z-VAD-fmk for 4 h and then subjected to MTT assay. **(D)** PARP-1, cleaved PARP-1, caspase-3 and cleaved caspase-3 expression was measured using western blot analysis in Hepa 1-6 cells treated with NTP-217 for 4 or 24 h. **(E)** Quantification of PARP-1, cleaved PARP-1, caspase-3 and cleaved caspase-3 expression. Data are presented as the mean ± SD (n=3). ^*^P<0.05, ^***^P<0.001, ^****^P<0.0001. ns, not significant; c, cleaved; Cas, caspase; PARP-1, poly (ADP-ribose) polymerase 1.

Since long incubation of cells with hybrid peptides could induce apoptosis, apoptosis-related experiments were performed. First, the caspase-3 activity assay demonstrated that 4-h treatment with NTP-217 did not activate caspase-3 on liver cancer cells, but treatment with NTP-217 for 24 h significantly increased the ratio of cells with activated caspase-3 (**Figures 3A, B**). Next, to further confirm that 4 h of treatment does not activate caspases, the pan caspase inhibitor z-VAD-fmk (20 μM) was co-applied with NTP-217 for 4 h. The results suggested that z-VAD-fmk did not reverse the cytotoxicity of NTP-217, and there was no significant difference between the dose-response curve of NTP-217 and that of NTP-217 plus z-VAD-fmk (**Figure 3C**). As shown in **Figures 3D, E**, regardless of the NTP-217 concentration, 4 h of treatment could not cause an increase in the expression of cleaved caspase-3 in Hepa 1-6 cell lines, whereas 24-h treatment remarkably enhanced the level of cleaved caspase-3. Moreover, poly (ADP-ribose) polymerase 1 (PARP-1), a substrate of cleaved caspase-3, was not cleaved with 4 h of treatment, whereas 24-h treatment markedly increased the level of cleaved PARP-1. In summary, treatment with NTP-217 for 4 h could not activate caspase-3, and the type of cell death induced by such a brief treatment was not apoptosis.

### Rapid necrosis of hepatoma cells induced by NTP-217

**Figure 4:**
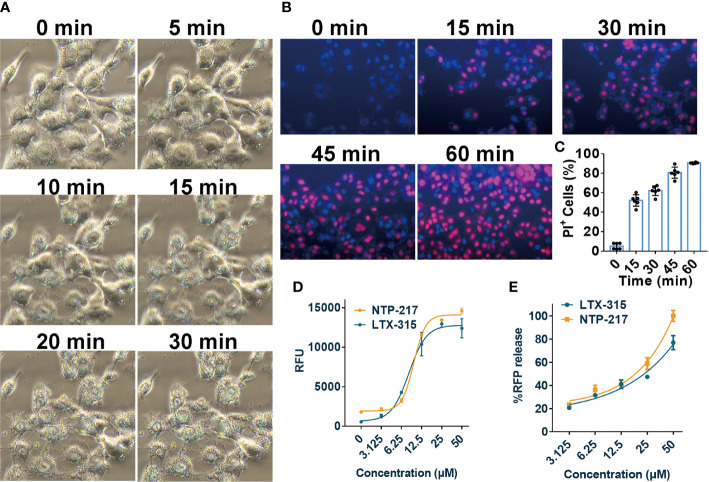
Induction of rapid necrosis of liver cancer cells by NTP-217. **(A)** Morphological changes of Hepa 1-6 cells after incubation with NTP-217 (25 μM) for different lengths of time (objective magnification, x20). **(B)** PI/Hoechst co-staining shows the loss of membrane integrity in 25 μM NTP-217-treated Hepa 1-6 cells (objective magnification, x10). **(C)** Quantification of PI-positive cells in panel B (n=6). The results are presented as the mean ± SD. **(D)** Levels of leaked ATP from Hepa 1-6 cells subjected to treatment with various concentrations of NTP-217 or LTX-315 for 4 h (n=3). **(E)** Fluorescent intensities of leaked RFP from Hep3B-Luc2-tdT cells subjected to treatment with various concentrations of NTP-217 or LTX-315 for 0.5 h (n=3). PI, propidium iodide.

The effects of NTP-217 on cell morphology, the ratio of PI-positive cells, and the degree of ATP and fluorescence leakage were investigated in peptide-treated cells. Under NTP-217 treatment, Hepa 1-6 cells showed a marked membrane rupture, with numerous membrane fragments dispersed in the culture medium (**Figure 4A**). The membrane integrity of NTP-217-treated Hepa 1-6 cells could be clearly observed by fluorescence imaging using nuclear dyes. Hoechst 33258 stains cell nuclei, while PI stains the nucleus of those cells whose membrane is not intact. As depicted in **Figures 4B, C**, 50% of the treated cells showed PI/Hoechst co-staining within 15 min, and cells incubated for 60 min with NTP-217 exhibited extensive PI incorporation into the nuclei (~90%). Moreover, the quantity of ATP released into the culture medium corresponded to the degree of loss of cell membrane integrity; thus, an ATP leakage assay was used employing Celltiter-LUMI™ luminescence detection reagent. NTP-217 caused ATP leakage in treated cells in a dose-dependent manner (**Figure 4D**). The fluorescence leakage assay employed a Hep3B cell line with a cytosol-expressed red fluorescent protein (Hep3B-Luc2-tdT). In this assay, the fluorescence intensity of the tdTomato released into the culture media corresponded to the extent of loss of cell membrane integrity. The results showed that NTP-217 induced fluorescence leakage in a concentration-dependent manner (**Figure 4E**). In summary, NTP-217 induced rapid necrosis characterized by membrane lysis and cytosolic components leakage in hepatoma cells.

### Mitochondrial leakage induced by NTP-217

**Figure 5:**
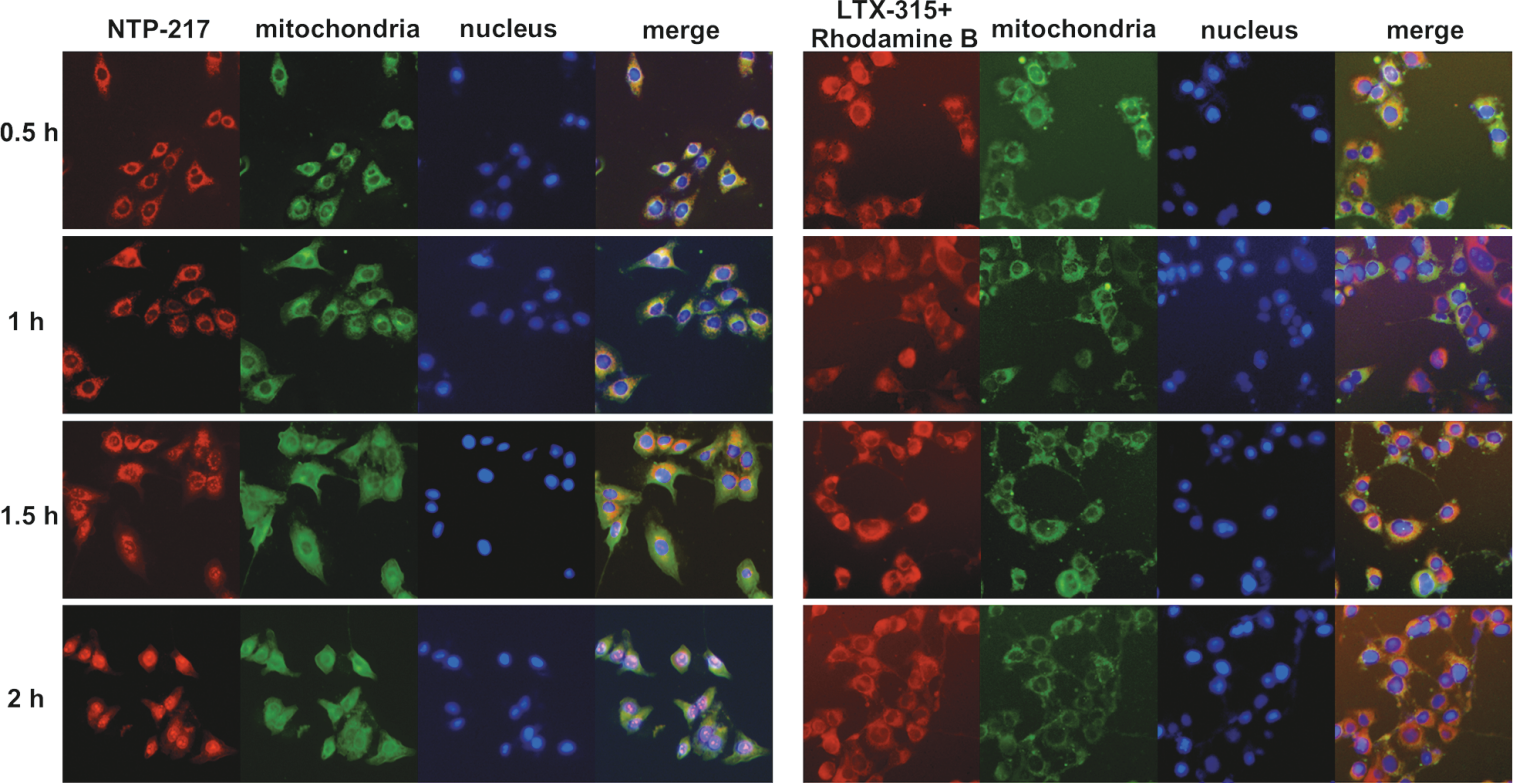
Induction of mitochondrial leakage by NTP-217. Fluorescent images of Hepa 1-6 cells incubated with 50 μM NTP-217 or 50 μM LTX-315 plus 50 μM rhodamine B for different lengths of time (objective magnification, x20). Mitochondria were labeled with Mito-Tracker Green, while nuclei were stained with Hoechst 33258.

To explore the differences in the mechanism of action between NTP-217 and LTX-315, imaging in living cells was carried out using NTP-217 or LTX-315 plus rhodamine B. In the presence of LTX-315, rhodamine B eventually accumulated into the mitochondria and was not detected in the cell nuclei. By contrast, NTP-217 only stayed for a short time in the mitochondria (~1 h) and was subsequently transferred into the cell nuclei (**Figure 5**). Notably, after 1 h of treatment with NTP-217, the distribution of green fluorescence (mitochondria marker) in the cytoplasm became more uniform, and over time, the bright green, fluorescent circle outside the cell nuclei gradually disappeared (**Figure 5**), which may be due to the fact that NTP-217 inflicted mitochondrial leakage and the Mito-Tacker Green labeled-substances within the mitochondria leaked into the cytoplasm. However, a similar phenomenon did not occur in cells treated with LTX-315 plus rhodamine B, demonstrating that NTP-217 was more destructive toward mitochondria than LTX-315.

### Loss of MMP and increase in ROS levels caused by NTP-217

**Figure 6:**
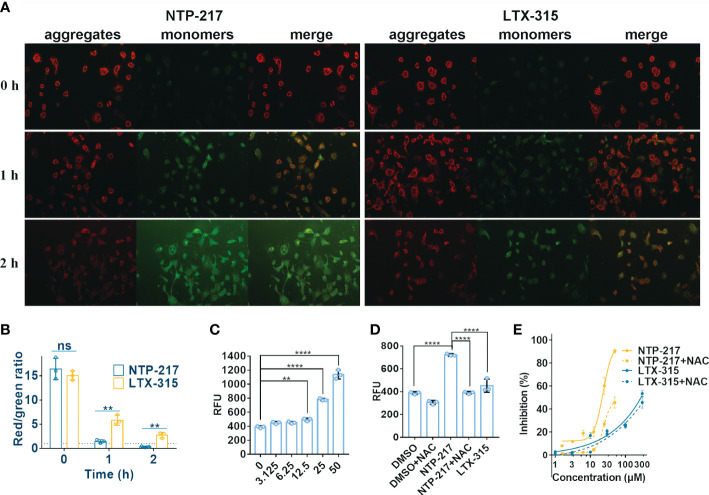
Decrease in mitochondrial membrane potential and increase in ROS level caused by NTP-217. **(A)** Hepa 1-6 cells treated with 25 μM NTP-217 or LTX-315 were subsequently labeled with JC-10 (objective magnification, x10). **(B)** Quantification of fluorescent intensity using ImageJ software (n=3). The results are presented as the mean ± SD. Student’s t-test. **(C)** ROS levels in Hepa 1-6 cells incubated with a series of concentrations of NTP-217 for 1 h were measured using fluorometric analysis (n=3). The data are presented as the mean ± SD. **(D)** ROS levels were measured in Hepa 1-6 cells upon incubation with DMSO, 25 μM NTP-217, DMSO plus 10 mM NAC, 25 μM NTP-217 plus 10 mM NAC or 25 μM LTX-315 (n=3). The data are presented as the mean ± SD. **(E)** Hepa 1-6 cells were incubated with NTP-217 alone or in combination with 10 mM NAC for 4 h and then subjected to MTT assay. Error bars indicate SD, and the results are representative of 3 independent experiments. ROS, reactive oxygen species; NAC, N-acetyl-L-cysteine. ^**^P<0.01, ^****^P<0.0001. ns, not significant.

The capacity of NTP-217 to provoke changes in the MMP was detected by using the fluorophore JC-10. This dye shows fluorescence emission in two typical colors: i) Red fluorescent aggregates at high mitochondrial potential, and ii) green, fluorescent monomers at low mitochondrial potential. **Figure 6A** revealed that an intense green fluorescence signal was observed in cells treated with 25 μM NTP-217 for 2 h and the ratio of red to green fluorescence was markedly reduced from 16.4 to 0.34 (**Figure 6B**), indicating that, over time, NTP-217 led to a remarkable reduction in MMP.

Since mitochondria are the main source of intracellular ROS, the ROS level was measured in the present study. NTP-217 increased ROS levels in a concentration-dependent manner in hepatoma cells (**Figure 6C**). The antioxidant N-acetyl-L-cysteine (NAC, 10 mM) could attenuate the NTP-217-induced increase in ROS levels (**Figure 6D**), and co-addition of NAC partly reversed the NTP-217-induced proliferation inhibition of liver cancer cells (**Figure 6E**), demonstrating that NTP-217-induced cytotoxicity in liver cancer cells was associated with ROS.

## Discussion

Liver cancer is an aggressive malignancy that is highly resistant to conventional systemic chemotherapy due to its heterogeneity and multiple etiologies (24). Hence, oncolytic peptides targeting cancer heterogeneity could be a better treatment choice (6). LTX-315 is a classic oncolytic peptide and previous studies have proved that intratumoral radiofrequency ablation (RFA)-associated radiofrequency hyperthermia can improve the efficacy of LTX-315 combined with doxorubicin in the treatment of liver cancer (25) and LTX-315 can prevent residual liver tumors after RFA (26). Moreover, LTX-315 has been evaluated in phase I/II studies (27). The present results demonstrated that LTX-315 exerted limited effects on liver cancer cells (IC_50_ >120 μM for 4 h), while the hybrid peptide NTP-217 obtained from the fusion of LTX-315 with rhodamine B increased anti-liver cancer activity by 4.8-8.7-fold relative to LTX-315. A similar result was found in animal experiments, where NTP-217 markedly inhibited hepatoma growth by 92.0% (tumor weight) and prolonged the overall survival of mice bearing tumors. By contrast, LTX-315 showed limited advantages in tumor growth inhibition, with a decrease in tumor weight of 65.6%. As early as 2016 the study has shown that LTX-315 caused the tumor disappearance in the rat hepatoma model (28). The present study did not exhibit that LTX-315 induced the complete disappearance of hepatoma, maybe because the administration time in the previous study was 7 days but 3 days in this study, but under the same condition, after the treatment with NTP-217 hepatoma disappeared completely. In order to easily monitor the reaction of the tumor toward peptides, a subcutaneous tumor model was used in this study, but the orthotopic animal models provided highly valuable clinical information than subcutaneous tumor models as the biological and metastatic microenvironment similar to clinical status. Hence, the tumoricidal effects of NTP-217 may need to be further examined in orthotopic hepatoma models. Living imaging showed that even after 48 h post-injection, the fluorescence response of NTP-217 in the tumor was still stable. However, this fluorescent response may show the rhodamine B molecule itself as a result of the degradation of NTP-217. Hence, the metabolic processes of NTP-217 need further study.

Cell imaging suggested that similar to LTX-315 targeting mitochondria (29), NTP-217 also accumulated in these organelles, although this accumulation was temporary. This brief accumulation led to the leakage of mitochondrial contents to the cytoplasm, which was indicated by the more uniform green fluorescence emitted by Mito-Tracker Green in the cytoplasm. Mitochondrial leakage dysregulated the functions of mitochondria and induced a decrease in MMP (30). It is widely accepted that mitochondria are the main source of intracellular ROS, and mitochondrial dysfunction is particularly prone to induce an increase in ROS levels in cells (13). NTP-217 enhanced ROS levels in a dose-dependent manner. Hence, this capacity to induce mitochondrial leakage answers the question of where the substantial ROS observed in the present study derived from. The mechanism by which NTP-217 leads to mitochondrial leakage is worth further investigating.

Prolonged treatment with hybrid peptides could induce ROS-mediated apoptosis (12). Whether short-time treatment with NTP-217 (4 h) could also induce apoptosis was investigated in the present study. The results of apoptosis-related experiments illustrated that treatment with NTP-217 for 4 h could not activate caspase-3, the executor of cell apoptosis, and a pan caspase inhibitor was not able to reverse NTP-217-induced cytotoxicity, which was in accordance with LTX-315 (18). Thus, the type of cell death induced by treatment with NTP-217 for 4 h was not apoptosis. Multiple studies have reported that the lead peptide LTX-315 can provoke necrotic cell death in cancer cells within 4 h (31, 32). Whether hybrid peptides can also induce necrosis was investigated in the present study. The results suggested that 4-h treatment with NTP-217 induced membranolysis, leakage of cytosolic components to the extracellular environment and ATP leakage in liver cancer cells, which are the typical features of necrotic cell death. Thus, it was concluded that hybrid peptides possessed dual anticancer effects on cancer cells: First, short-time treatment with hybrid peptides could trigger the rapid necrosis of cancer cells. If rapid necrosis was not sufficient to kill cancer cells, thanks to their nucleus-targeting capacity, long-time treatment with hybrid peptides could also damage cell nuclei and induced ROS-mediated DNA DSB-associated intrinsic apoptosis (12). Although different forms of cell death were induced by hybrid peptides at different time scales, the mechanism of transition from necrosis to apoptosis needs further investigation.

In summary, the present results demonstrated the rapid and superior therapeutic effect of NTP-217 on liver cancer and suggested mitochondrial leakage and ROS generation to be the possible mechanisms by which NTP-217 increased LTX-315-induced proliferation inhibition *in vitro* and exhibited anticancer efficacy *in vivo*. However, further studies, including toxicity, accumulation and degradation in the body and clinical trials, are required to fully support the use of hybrid peptides as a strategy for the therapy of hepatoma.

## Data availability statement

The original contributions presented in the study are included in the article/**Supplementary Material**. Further inquiries can be directed to the corresponding authors.

## Ethics statement

The animal study was reviewed and approved by Medical Ethics Committee of the Affiliated Hospital of Qingdao University.

## Author contributions

HY and YKQ conceived the project, designed the experiments, and analyzed and interpreted the data. YKQ and SSD designed oncolytic peptides. HY performed most experiments with the assistance of XYF and HYG. XYF, YNM and XTC synthesized peptides. HY, YKQ, HG and XQZ wrote the manuscript. YKQ and SSD prepared the article. All authors contributed to the article and approved the submitted version.
