# Supplementary material for: Hybrid peptide NTP-217 triggers ROS-mediated rapid necrosis in liver cancer cells by induction of mitochondrial leakage
Source: Front Oncol. 2023 Jan 12;12:1028600. doi: 10.3389/fonc.2022.1028600 (PMC9881410; doi:10.3389/fonc.2022.1028600)
Supplement: Supplementary file 1 [file DataSheet_1.docx]

Supplementary Material

# Supplementary Figures

**Supplementary Figure 1.** The molecular structures of NTP-217 (top) and LTX-315 (bottom).

**Supplementary Figure 2.** Analytical RP-HPLC and ESI-MS spectra of the purified peptide NTP-217.
